# Red urine in children

**DOI:** 10.1186/1824-7288-40-S1-A19

**Published:** 2014-08-11

**Authors:** Carmine Pecoraro, Ilaria Luongo

**Affiliations:** 1 Unit of Nephrology and Dialysis, Children's Hospital "Santobono" via M.Fiore,6 (80129) Naples, Italy

## 

Red Urine (RU) is an uncommon finding in an unselected population of children; its prevalence was reported as 0.13 % based on a retrospective USA review of children. However, RU may signify disorders ranging from benign conditions such as medications and food coloring to more serious conditions such as urinary tract infection, hereditary cystic renal diseases and glomerulonephritis. In the clinical approach and management of these children, it is important to firstly differentiate true gross hematuria from non-gross hematuria, and subsequently dissecting out the different causes of gross hematuria (GH).

To determine the prevalence and the etiology of GH in an italian general pediatric setting we undertook a prospective study of all patients with GH in pediatric emergency walk-in clinic for consecutive 15 months. Study protocol: all patients who complained of RU were referred to the pediatric nephrologist for complete evaluation until diagnosis was made. Over the 15-month period 82,934 children visited the Emergency Clinic at our Children’s Hospital; 85 patients made visit in which they complained of RU (1.1/ 1,000 visits). Sex ratio showed a male preponderance (M 50/F 35); mean age was 7.06 ± 4.6 (range 0.5-16.1 years). Among diagnosis, glomerulopathies accounted for 59.5% : Post infectious acute glomerulonephritis (37), primary (7) and secondary to Schonlein-Henoch purpura (3) IgA nephropathy (10), SLE (1), Alport Syndrome (3). 14/85 patients with GH had UTI ( 12 documented bacterial UTI and 2 with a presuntive adenovirus cystitis); 16.5% had Idiopathic Hypercalciuria (6 out showed microcalculi at ultrasonography); 2 patients suffered from hyperuricosuria; 1 child was affected by haemolytic uremic syndrome, 2 myoglobinuria from rabdomyolisis and in 1 case no diagnosis was made. Our prospective study indicates that in our country, according to the previous report, the prevalence of GH is relatively uncommon, but the most common causes are represented by hematuric glomerulonephritis (mainly PIAGN and IGAN) in boys, and not by proven (26%) and unproven (23%) UTI mainly in girls; just one child was categorized as “unknown etiology” vs 9% of USA children. Two main reasons may explain such a difference: 1) the health social assurance systems, namely the availability in Italy of the family pediatrician which avoid that children with GH from trivial causes such as hemorragic cystitis, will turn to ER and 2) the systematic evaluation of all cases of GH, in our experience, directly by the paediatric nephrologist who could assure a definite diagnosis.

